# Molecular Characterization, Evolution, and Expression Profiling of the *Dirigent* (*DIR*) Family Genes in Chinese White Pear (*Pyrus bretschneideri*)

**DOI:** 10.3389/fgene.2018.00136

**Published:** 2018-04-16

**Authors:** Xi Cheng, Xueqiang Su, Abdullah Muhammad, Manli Li, Jinyun Zhang, Yanming Sun, Guohui Li, Qing Jin, Yongping Cai, Yi Lin

**Affiliations:** ^1^School of Life Science, Anhui Agricultural University, Hefei, China; ^2^Institute of Horticultural, Anhui Academy of Agricultural Sciences, Hefei, China

**Keywords:** *Dirigent* family, *Pyrus bretschneideri*, phylogenetic analysis, expression analysis, lignin synthesis, stone cell

## Abstract

Stone cells content and size are the key factors determining the internal quality of the pear fruit. Synthesis of lignin and thickening of secondary cell wall are the keys to the development of stone cells. The polymerization of monolignols and secondary cell wall formation requires the participation of dirigent proteins (DIRs). In recent years, *DIR* family have been studied in higher plants, but lack of comprehensive study in the pear *DIR* (*PbDIR*) family. This study focuses on the identification and analysis of *PbDIR* family for the first time. We identified 35 *PbDIR*s from the pear genome, 89% of which are intronless genes. Phylogenetic tree and chromosome localization analysis showed that 35 *PbDIR*s were divided into four subfamilies (DIR-a, -b/d, -e, and -g) and irregularly distributed among 10 chromosomes. In addition, we identified 29, 26, and 14 DIRs from the other three Rosids (peach, Mei, and grape), respectively. Interspecies microsynteny analysis revealed the collinear gene pairs between pear and peach are the most. Temporal expression analysis showed that the expression changes of seven *PbDIR*s (DIR-a subfamily: *PbDIR4* and *PbDIR5*; DIR-b/d subfamily: *PbDIR11*; DIR-g subfamily: *PbDIR19*; DIR-e subfamily: *PbDIR23, 25* and *26*) in fruits were consistent with the changes of fruit lignin and stone cells contents. In addition, the subfamily of *PbDIR*s in fruits showed significant responses after treatment with ABA, SA, and MeJA. According to the protein tertiary structure, key amino acid residues and expression patterns analysis found that PbDIR4 might be involved in the metabolism of lignin and related to stone cells contents in pear fruits. In this study, we systematically analyzed the structure, evolution, function and expression of *PbDIR* family, which not only confirmed the characteristics of *PbDIR* family, but also laid the foundation for revealing the role of *DIR* in pear stone cell development and lignin polymerization.

## Introduction

Dirigent proteins (DIRs) were first found in *Forsythia* X *intermedia* (Davin et al., 1997) and were subsequently studied in other plants. DIRs are found in almost all vascular plants (Davin and Lewis, 2000), which often in the form of the gene family. At present, 25, 54, 35, 9, 29, and 19 *DIR*s have been found in *Arabidopsis thaliana, Oryza sativa, Picea, Thuja plicata, Brassica rapa*, and *Isatis indigotica* (Ralph et al., 2007; Thamil Arasan et al., 2013; Li et al., 2014; Paniagua et al., 2017). Ralph et al. (2007) classified the members of the *DIR* family into six subfamilies: DIR-a, DIR-b/d, DIR-c, DIR-e, DIR-f, and DIR-g subfamilies. Biochemical experiments show that the members of DIR-a subfamily can direct the polymerization of monolignols into the correct three-dimensional structure, and the functions of other subfamily members are unknown. Therefore, the *DIR* that except DIR-a subfamily is called the *DIR-like* (Davin et al., 1997; Xia et al., 2000; Kim et al., 2002; Ma and Liu, 2015).

The dirigent protein model hypothesis suggests that the formation of lignin oligomers is carried out under the strict regulation of DIRs, which controls the formation of specific chemical bonds during the monolignol polymerization process to form lignin polymers (Vanholme et al., 2012; Barros et al., 2015; Paniagua et al., 2017). Furthermore, *in situ* mRNA hybridization and immunolabeling techniques showed that lignin synthesis related DIRs were mainly distributed in the secondary cell wall and other lignification tissues (Davin and Lewis, 2000; Burlat et al., 2001; Yin et al., 2013; Dima et al., 2015). Therefore, DIRs are closely related to lignification of plant cells and plays an important role in secondary cell wall formation. In addition, studies have shown that DIR can also catalyzed polymerization of monolignol (coniferyl alcohol) to form lignans, partial synthetic pathway of lignin and lignan are shared. Lignan can directly transport to the cell wall and converted into lignins (Yin et al., 2013). DIRs improve plant stress resistance by regulating the metabolism of lignin and lignan (Thamil Arasan et al., 2013; Ma and Liu, 2015; Li et al., 2017b; Paniagua et al., 2017). Therefore, DIRs plays an important role in the process of plant stress resistance.

Pear is one of the three temperate fruit species, of which ‘Dangshan Su’ pear (*Pyrus bretschneideri* cv. Dangshan Su) origin in Dangshan County, Anhui Province, China. ‘Dangshan Su’ pear has a long history of cultivation, is the diploid pear varieties with largest cultivated area and occupies an important position in China and even around the world (Cheng et al., 2017; Zhang et al., 2017). However, the stone cell content is one of the main defects of this variety. Stone cell is a special cell of pear, it is a kind of dead cell formed by cell wall lignification of parenchyma cells in the flesh. If the content is too much, it will lead to the occurrence of hard-end disorder or stoney pit of pear (Lu et al., 2015). Eventually make the fruit taste rough, nutrients decreased, which seriously affects the quality and economic benefits of pear (Jinho et al., 2007; Cai et al., 2010; Choi and Lee, 2013; Li et al., 2017a). Therefore, the regulation of the stone cell content is the key to improve the quality of ‘Dangshan Su’ pear.

In recent years, a large number of studies have proved that the stone cells of pear contain approximately 30% lignin (Brahem et al., 2017; Zhang et al., 2017). During the development of stone cells, the secondary wall of the stone cell is thickening and a large amount of lignin is deposited in secondary cell wall until the whole cell is filled. Therefore, lignin is not only one of the major components of stone cells, but also the polymerization and deposition of lignin is essential for its development (Nii et al., 2008; Jin et al., 2013; Zhao et al., 2013; Yan et al., 2014). The regulation of lignin polymerization and deposition is an effective means to reduction the content of stone cells in pear fruit.

At present, there are few studies on the pear *DIR* (*PbDIR*) family, and the functions of each subfamily members are not clear. In this study, the bioinformatics method was used to identification *PbDIR*s from the pear genome, and the classification, chromosome distribution, conserved motifs, gene structures and evolutionary characteristics of each member were analyzed. Meanwhile, temporal expression and hormone response patterns were also analyzed. In order to explore the basic characteristics of the *PbDIR* family, predict the function of *PbDIR*s and screen out the candidate members related to the lignin metabolism of pear fruit. This can provide the target gene for further study of lignin monomer polymerization in pear fruits and lay the foundation for regulating pear lignin metabolism and stone cell development.

## Materials and Methods

### Plant Materials

**FIGURE 1:**
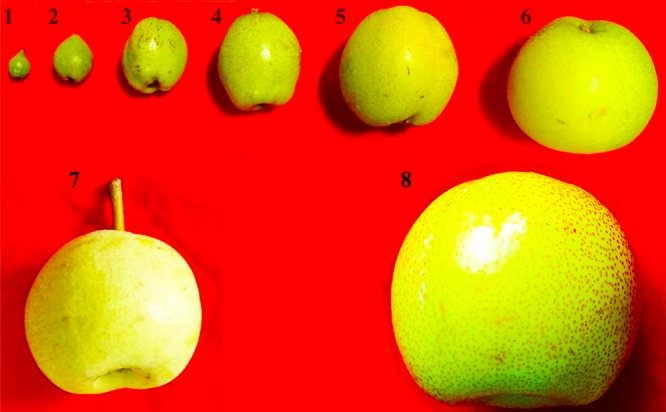
Observation on the phenotypes of eight fruit developmental stages of pear. 1–8: 15, 39, 47, 55, 63, 71, 102, and 145 days after flowering (DAF).

Fruits were obtained from 50-year-old ‘Dangshan Su’ pear trees grown on a farm in Dangshan County, Anhui Province, China. We collected fruits at 8 development stages: 15 days after flowering (DAF), 23, 39, 47, 63, 79, 102, and 145 DAF (maturity) (**Figure 1**). Sixty pears were collected at each development stage, and the samples were stored at -80°C until use. In the case of exogenous hormone treatments, the 0.5 mmol/L abscisic acid (ABA), 0.5 mmol/L methyl jasmonate (MeJA), or 0.2 mmol/L salicylic acid (SA) was sprayed onto fruits at 39 DAF.

The total RNA was isolated from each sample using a total RNAprep Pure Plant Kit (Tiangen, China). Reverse transcription was performed using a EasyScript One-Step gDNA Removal and cDNA Synthesis SuperMix (TransGen Biotech, China).

### Identification and Classification of *DIR* Family Members in Genome

*DIR* family members were identified in four species genome. *Pyrus bretschneideri* (pear) and *Prunus mummer* (Mei) genome files were downloaded from the GigaDB data^1^. The genome information of *Prunus persica* (peach) and *Fragaria vesca* (strawberry) were obtained from Phytozome database^2^. The conserved domain (PF03018) of *DIR* family was used as target sequence to screen the candidate *DIR* family members from the genome databases using the Blastp (*E* = 1.0E-10) function of the BioEdit software. After repeated redundant sequences were removed, Pfam^3^ was used to identify whether the candidate sequence contained the conserved domain of DIR. The sequences with DIR conserved domain were the *DIR* family members. The sequence characteristic information are also further analyzed. The isoelectric point (pI) and molecular weight (MW) of each DIR was predicted using ProtParam3^4^, subcellular localization was analyzed using WoLFPSORT^5^. The amino acid length and chromosome information are available from the genomic file.

### Phylogenetic Analysis and Protein Structure Prediction

The phylogenetic trees were constructed using the maximum likelihood (ML) (bootstrap = 1000) in MEGA5.1 software (Tamura et al., 2011). The gene sequence information used to construct the phylogenetic tree is listed in Supplementary Table S1. The secondary and three-dimensional structures of proteins were predicted by Espript3.0^6^ and Protein Fold Recognition Server^7^, respectively.

### Exon–Intron Structures and Conservative Motifs Prediction

The exon–intron structures of *PbDIR*s were generated using GSDS^8^. The conserved motifs were confirmed using Multiple Em for Motif Elicitation (MEME)^9^. The specific setting parameters are as follows: Motif width is greater than 6 and less than 200. A total of 20 motifs were identified.

### Collinearity Analysis

Collinearity analysis was completed using Plant Genome Duplication Database (PGDD) (Lee et al., 2013) and circos software^10^.

### Chromosomal Locations, Gene Duplications and *K*_a_/*K*_s_ Ratios Analysis

The chromosomal locations of each member are generated using MapInspect software^11^, and the specific location information of each member is obtained from the pear genome database.

According to previously reported method, this study defines criteria for determining gene duplication events is: the match lengths of the two coding sequence (CDS) that match each other are greater than 80% of the length of the longer sequences, and the similarity of the two gene sequences to each other is greater than 80%. If a pair of duplication genes is on the same chromosome and the number of genes between the two given genes is less than 5, they are tandem duplicates; otherwise, they are segmental duplicates (Ouyang et al., 2009; Kong et al., 2013; Wei et al., 2013).

DnaSP v5.0 software was used to calculate the *K*_a_ (non-synonymous substitution rate) and *K*_s_ (synonymous substitution rate) of each duplication gene pair (Rozas and Rozas, 1995). According to the study of Kong et al. (2013) and Wei et al. (2013), if *K*_a_/*K*_s_ > 1, there is a positive selection effect, if *K*_a_/*K*_s_ = 1, there is a neutral selection, *K*_a_/*K*_s_ < 1, there is purifying selection effect. Sliding window analysis was also performed using this software.

### *In Silico* Analysis of *PbDIR*s 5′ Upstream Regions

We searched the 1500 bp 5′ upstream sequence from each *PbDIR* initiation codon and analyzed the type and number of *cis*-acting regulatory DNA elements (*cis*-elements) using the PLANT CARE program^12^.

### qRT-PCR Analysis for *PbDIR*s

qRT-PCR was performed using the CFX96 Touch^TM^ Real-Time PCR Detection System (Singapore). Pear *Tubulin* gene (Genbank accession No. AB239680.1) (Wu et al., 2012) was used as an internal reference, and the relative expression levels of the genes were calculated using the 2^-ΔΔC_T_^ method (Livak and Schmittgen, 2001). Each reaction was performed in triplicate. The primer sequences are listed in Supplementary Table S2. Genes expression profile was visualized using HeatmapCluster software^13^. The values were *Z*-score transformed according to the column.

## Results

### *PbDIR*s Identification, Characterization and Analysis

We identified 35 *DIR* family members in the pear genome with conserved domains (PF03018), respectively, named *PbDIR1*-*PbDIR35* (**Table 1**). It was found that the amino acid length of 35 *PbDIR* family members was mainly between 85–391 aa and the average length was about 211 aa. The MW of PbDIRs was between 9.35–40.37 kDa and the average MW was about 22.53 kDa.

**Table 1:** Sequence information of 35 PbDIRs identified.

Name	Sequence ID	Chr	Protein			
			Length (aa)	MW (kDa)	pI	Subcellular localization
*PbDIR1*	Pbr003341.1	11	193	21.37	9.07	Chloroplast
*PbDIR2*	Pbr000713.1	3	129	14.38	6.25	Cytoplasm
*PbDIR3*	Pbr003340.1	11	194	21.38	8.96	Chloroplast
*PbDIR4*	Pbr024647.1	6	185	20.90	7.05	Cytoplasm
*PbDIR5*	Pbr037951.1	2	190	20.47	9.75	Chloroplast
*PbDIR6*	Pbr008887.1	9	196	21.40	9.16	Chloroplast
*PbDIR7*	Pbr034394.1	14	182	19.73	7.99	Chloroplast
*PbDIR8*	Pbr034396.1	14	153	16.67	8.83	Cytoplasm
*PbDIR9*	Pbr022957.1	2	148	16.13	5.04	Cytoplasm
*PbDIR10*	Pbr041181.1	5	190	20.86	9.19	Extracellular
*PbDIR11*	Pbr009712.1	7	204	22.36	5.91	Chloroplast
*PbDIR12*	Pbr031630.1	5	171	18.85	8.05	Cytoplasm
*PbDIR13*	Pbr041180.1	5	193	20.93	7.82	Chloroplast
*PbDIR14*	Pbr041182.1	5	192	20.64	9.30	Chloroplast
*PbDIR15*	Pbr021616.1	10	183	20.48	9.66	Nucleus/Cytoplasm
*PbDIR16*	Pbr007174.1	14	180	19.47	8.84	Chloroplast
*PbDIR17*	Pbr022959.1	2	180	20.05	9.26	Chloroplast
*PbDIR18*	Pbr041809.1	9	175	18.93	7.85	Chloroplast
*PbDIR19*	Pbr005147.1	9	183	20.51	6.50	Chloroplast/Vacuolar
*PbDIR20*	Pbr009711.1	7	85	9.35	9.22	Cytoplasm
*PbDIR21*	Pbr033975.1	3	310	31.96	5.22	Chloroplast
*PbDIR22*	Pbr032312.1	7	301	31.34	4.76	Cytoplasm
*PbDIR23*	Pbr032311.1	7	391	40.37	4.63	Chloroplast
*PbDIR24*	Pbr023468.1	11	310	32.23	5.22	Chloroplast
*PbDIR25*	Pbr038160.1	/	228	23.78	4.77	Nucleus/Cytoplasm
*PbDIR26*	Pbr026460.1	7	389	40.15	4.58	Chloroplast
*PbDIR27*	Pbr038161.1	/	306	31.84	4.81	Chloroplast
*PbDIR28*	Pbr021601.1	10	95	10.61	8.20	Nucleus/Extracellular
*PbDIR29*	Pbr041290.1	9	245	25.09	5.40	Extracellular
*PbDIR30*	Pbr041289.1	9	252	25.78	4.97	Extracellular
*PbDIR31*	Pbr021145.1	4	305	31.82	4.81	Extracellular
*PbDIR32*	Pbr012775.1	2	134	14.55	7.93	Chloroplast
*PbDIR33*	Pbr021146.1	4	256	27.15	4.82	Nucleus
*PbDIR34*	Pbr009710.1	7	101	11.20	9.60	Cytoplasm
*PbDIR35*	Pbr032310.1	7	239	25.74	6.79	Chloroplast

*“/” indicates that gene could not be conclusively mapped to any chromosome*.

The pI of PbDIRs with large variable range (4.58–9.75), of which a total of 19 members pI is alkaline (pI > 7.0). Subcellular localization prediction showed that PbDIRs were mainly located in chloroplast, with nineteen members (54%), among which PbDIR19 was located in chloroplast and vacuolar. There are 10 members located in cytoplasm, in which PbDIR15 and PbDIR25 are simultaneously located in the nucleus and cytoplasm. In addition, there are five members located in extracellular, but PbDIR28 are also distributed in the nucleus. PbDIR33 is only located in the nucleus.

**FIGURE 2:**
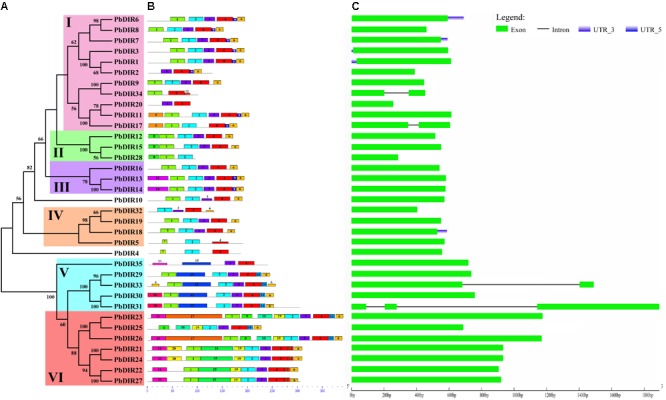
The conserved motifs and exon–intron structures of *PbDIR* family members based on the evolutionary relationship. **(A)** Phylogenetic relationships of PbDIRs. **(B)** Distribution of 20 putative conserved motifs in PbDIRs. **(C)** Gene structures of *PbDIR* family members.

We constructed a phylogenetic tree of 35 PbDIRs to further clarify the evolutionary relationship between PbDIRs (**Figure 2A**). It was found that 35 *PbDIR*s in the pear genome formed 12 gene pairs. These PbDIRs can be roughly divided into I–VI six subfamilies, in which the subfamily I contains the largest number of members with 11 PbDIRs, followed by the subfamily VI with seven members. The subfamilies IV and V contains four and five members, respectively. The subfamilies II and III are all composed of three PbDIRs. In addition, PbDIR4 and 10 are not clustered into the six subfamilies, they each formed their separate clades.

### Conservative Motifs and Exon–Intron Structures Analysis of *PbDIR* Family Members

In order to understand the characteristics of *PbDIR* family deeply, we used MEME software and GSDS website to analyze the conservative motifs and exon–intron structures of 35 *PbDIR* family members (**Figure 2**).

First of all, 20 motifs of the *PbDIR* family members were identified via MEME software. And then SMART and Pfam software were used to perform annotation on each motif. It was found that motif 2 and 4 constitute dirigent domains, motif 5, 10, 12, and 16 constitute transmembrane region, and other motifs do not have annotation information for the moment (Supplementary Figure S1). Notably, all the PbDIRs contain these two motifs (motif 2 and 4) except for PbDIR2, 20, 28, 34, and 35. PbDIR2, 20, 34, and 35 only contain motif 4. PbDIR28 only contain motif 2. Therefore, although the dirigent domain of some PbDIRs is exhibited deficiency, all members containing the domain, indicating that the results of the screening are reliable.

The distribution of 20 motifs in each subfamily with a degree of regularity (**Figure 2B**). For example, motif 9 is only present in members of the subfamilies I and III; motif 10 and 13 are only present in members of the subfamily V; motif 14 is only present in the members of the subfamilies V and VI; motif 11, 20, and 17 are present in members of the subfamily VI; only PbDIR4 and 5 contain motif 7; motif 16 is only found in members of subfamily III. These results indicated that PbDIRs clustered into same subfamily showed similar motif kinds and distributions, suggesting these characteristic motifs may confer different functions for different subfamilies.

It can be seen from **Figure 2C** that the gene structure of *PbDIR*s is relatively simple, most of the members belong to the intronless genes except for *PbDIR17, 31, 33*, and *34*. And the *PbDIR*s with no intron in the pear accounted for 89%. There is a similar situation in *Arabidopsis* and rice. Generally, the exon length of the members in the same evolution branch is similar, which is consistent with the genetic relationship between them and further supports the clustering results of the phylogenetic tree.

### Chromosome Distribution, Gene Duplication Events and Functional Differentiation of *PbDIR*s

In order to clarify the distribution of *PbDIR*s on 17 chromosomes of pear, MapInspect software was used to map *PbDIR*s on the chromosome (Supplementary Figure S2). It can be found that except for *PbDIR25* and *PbDIR27* have not been mapped on specific chromosomes, the remaining 33 *PbDIR*s are irregularly distributed on chromosomes 2–7, 9–11, and 14. The *PbDIR*s on chromosomes 5 and 7 were all in the form of more obvious gene clusters.

Subsequently, we further identified the duplication gene pairs of the *PbDIR* family to understand the duplication events of *PbDIR*s in the pear genome (Supplementary Table S3 and Supplementary Figure S3). According to the criteria described in the method, we identified four pairs of duplication genes, *PbDIR13*/*PbDIR14* (tandem duplication), *PbDIR21*/*PbDIR24* (segmental duplication), *PbDIR23*/*PbDIR26* (tandem duplication), *PbDIR30*/*PbDIR31* (segmental duplication), respectively. It was found that the *K*_a_/*K*_s_ ratios of these four pairs of duplication gene were less than 1, indicating that they had a selective effect in the evolutionary process, and their function did not produce significant differentiation.

### Microsyntenies Were Identified in Four Plant Species

In order to analysis collinearity of *DIR* family in different species better, we also identified the *DIR* family members in the *P. persica, P. mume*, and *Vitis vinifera* genome, and their sequence properties, evolutionary relationship, gene structures and conserved motifs were analyzed (Supplementary Table S4 and Supplementary Figure S4). Similar to pear, *Arabidopsis* and rice, a large number of members in *PpDIR, PmDIR*, and *VvDIR* family also have no introns. It is presumed that no introns are more likely to be a characteristic of the *DIR* family.

**FIGURE 3:**
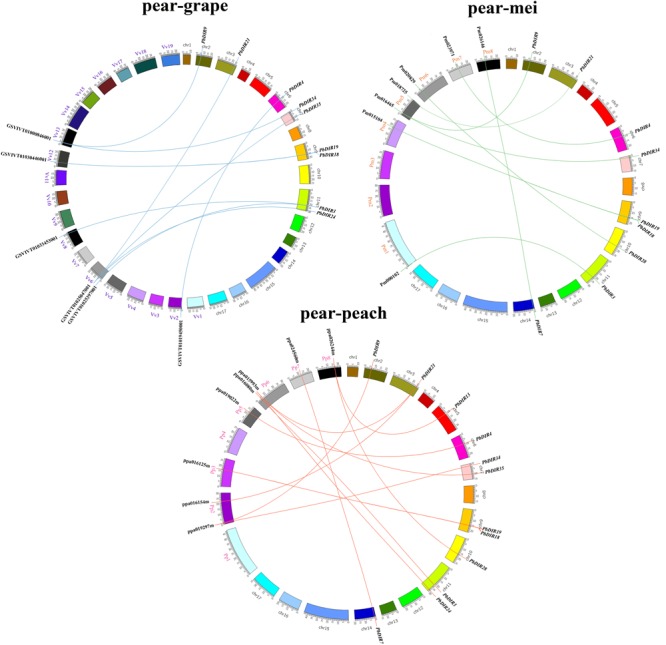
Extensive microsynteny of *DIR* regions across among *Prunus persica, Prunus mume*, and *Vitis vinifera*. Pp, *Prunus persica*; Pm, *Prunus mume*; Vv, *Vitis vinifera*.

Pear, peach, Mei and grape are all belong to the Rosids, which all originated from a common ancient hexaploid ancestor (Bremer et al., 2009). For the *DIR* family, 32 collinear gene pairs were found among pear, peach, Mei, and grape (**Figure 3**). There are 13 collinear gene pairs between pear and peach, 9 pairs between pear and Mei, 10 pairs between pear and grape. It is presumed that the pear and peach may have a closer relationship. It is worth noting that some *PbDIR*s also have a collinear relationship with the members of *PpDIR, PmDIR*, and *VvDIR* family, such as *PbDIR3* (Pbr003340.1), *PbDIR34* (Pbr009710.1), *PbDIR19* (Pbr005147.1), and *PbDIR9* (Pbr022957.1). This indicated that these gene pairs have already appeared before the divergence of common ancestor of pear, Mei, peach, and grape. Interestingly, some *PbDIR*s (*PbDIR28*, Pbr021601.1; *PbDIR7*, Pbr034394.1) only have the collinear relationship with the *PpDIR*s and *PmDIR*s, and the *VvDIR*s does not, indicating that these genes pairs formed after grape diverged from the common ancestor of pear, peach and Mei. In addition, there is a collinear relationship between one *PbDIR* and several *VvDIR*s or *PpDIR*s at the same time. For example, GSVIVT01025397001 and GSVIVT01033453001 are orthologous genes to *PbDIR3* (Pbr003340.1), ppa015993m and ppa016154m are orthologous genes to *PbDIR21* (Pbr033975.1), suggesting that they probably belong to paralogous gene pairs.

### Identification of *Cis*-Acting Elements in *PbDIR*s 5′ Upstream Regions

**FIGURE 4:**
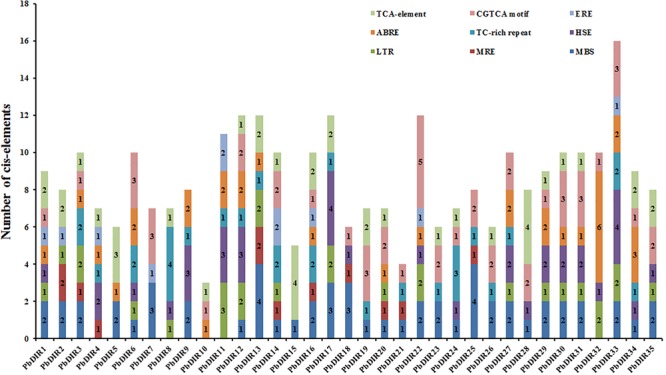
*Cis*-acting elements analysis of *PbDIR*s.

A large number of studies have shown that DIR is related to plant stress response. Therefore, the PLANT CARE program was used to identify the types and numbers of *cis*-acting elements involved in biotic and abiotic stress in 35 *PbDIR*s 5′ upstream regions (**Figure 4** and Supplementary Table S5). It is found that ten members of the *PbDIR* family contain MRE (light responsive element), indicating that the expression of these members is regulated by light. And, the 5′ upstream regions of *PbDIR*s also have more abiotic stress related elements such as MBS (drought), LTR (low-temperature) and HSE (heat). A total of 31 members contained MBS elements related to drought stress, and the number of this element in the *PbDIR* family was the largest, indicating that *PbDIR* was closely related to pear drought stress.

In addition, CGTCA motif, TCA-element, ABRE and ERE elements related to the hormone response of MeJA, SA, ABA, ethylene were also identified in the 5′ upstream regions of *PbDIR*s. In which, the number of members with ERE is the least, the total number of ERE elements in the 5′ upstream regions is also the least, suggesting that *PbDIR*s may play a minor role in plant maturation. A total of 26 members 5′ upstream regions contain 51 CGTCA motifs, 24 members contain 40 TCA-element. It is suggested that *PbDIR*s plays an important role in plant stress response, and may be able to resist stress by promoting lignin and lignans synthesis in plants.

### Classification and Functional Prediction of PbDIRs

In order to further clarify the phylogenetic relationship between PbDIRs and DIRs of other species, and to predict possible biological functions of PbDIRs. We construct a phylogenetic tree including *DIR* family members from pear, *Arabidopsis*, rice, *I. indigotica* and other function known DIRs.

**FIGURE 5:**
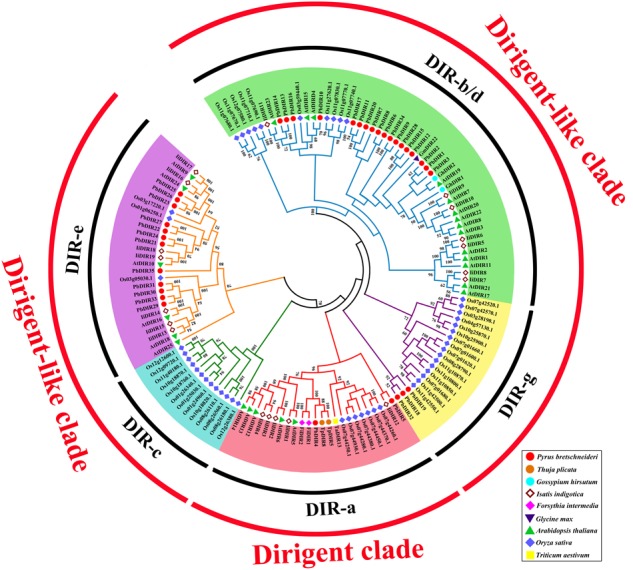
Phylogenetic tree constructed with PbDIRs and DIRs of other plant species. DIR sequences were aligned and a phylogenetic tree was constructed by ML using MEGA5.1. DIR-a, -b/d, -c, -e, and -g are shaded red, green, blue, purple, and yellow.

The 136 DIRs are classified according to the method of Ralph et al. (2007). It can be found from the **Figure 5** that these 136 DIRs can be divided into two clades (dirigent clade and dirigent-like clade) and five subfamilies (DIR-a, -b/d, -c, -e, and -g). There are 35 PbDIRs unevenly distributed in the four subfamilies (DIR-a, -b/d, -e, and -g); where subfamily DIR-a contains PbDIR4 and 5; subfamily DIR-b/d contain 18 PbDIRs (PbDIR1, 2, 3, 6, 7, 8, 9, 10, 11, 12, 13, 14, 15, 16, 17, 20, 28, and 34); subfamily DIR-e consists of 12 members (PbDIR21, 22, 23, 24, 25, 26, 27, 29, 30, 31, 33, and 35); subfamily DIR-g with three PbDIRs, PbDIR18, 19, and 32. Notably, there are none of the subfamily DIR-f members are present in these species. According to the present reports, the subfamily DIR-f members are only present in *Picea* spp. (Ralph et al., 2007). In addition, the clustering style of *DIR* family members from *I. indigotica, Arabidopsis* and rice in the phylogenetic tree was consistent with that previous reported, which proved that the phylogenetic tree classification of this study was reliable (Ralph et al., 2006, 2007; Li et al., 2014; Liao et al., 2017).

Members of DIR-a are considered bona fide DIRs, which play an important role in monolignols dimerization. The subfamily DIR-a members derived from dicotyledonous plants (*Arabidopsis, Isatis*, pear, and *Forsythia*), monocotyledonous plants (wheat, rice) and gymnosperms (*T. plicata*). AtDIR5, AtDIR6, FiDIR1, TaDIR13, TpDIR5, and TpDIR8 are all classified as this subfamily, and these proteins have been identified by the crystal structure or *in vitro* substrate catalysis experiments, and they are responsible for the coupling of lignin monomer (coniferyl alcohol) to form dimers (Kim et al., 2015; Ma and Liu, 2015; Gasper et al., 2016; Paniagua et al., 2017). PbDIR4 and PbDIR5 are both classified into this subfamily, it is speculated that these two PbDIRs play a similar role in pear.

DIR-b/d contains the largest number of members, and some studies have shown that most of the members of the subfamily respond to biotic or abiotic stress, participate in plant stress-resistant physiological processes (Damaj et al., 2010; Guo et al., 2012). Among them, GmDIR22 has been shown to be involved in the biosynthesis of pinoresinol (the dimer of coniferyl alcohol) and the overexpression of GhDIR1 can increase lignin content in cotton. These two genes are also related to plant resistance (Shi et al., 2012; Li et al., 2017b). Therefore, PbDIR1, 2 and 3 (which has a close relationship with them) may have similar biological functions to them, which is related to the stress response of pear and directly or indirectly participates in the metabolic process of lignin and pinoresinol. The members of the DIR-c were only present in monocotyledonous plants (Ma and Liu, 2015). In this study, subfamily DIR-c consisted of 13 rice DIRs, consistent with the results of Liao et al. (2017), but their function is not clear.

There are few studies on the function of DIR-e and DIR-g subfamilies, and only AtDIR10 in DIR-e has been shown to be related to the development of lignin-forming Casparian strips (Hosmani et al., 2013; Paniagua et al., 2017). AtDIR9, 10, 16, 18, 24, and 25 of this subfamily were highly expressed in roots of *Arabidopsis*, but no significant change patterns were observed in biotic or abiotic stresses (Paniagua et al., 2017). However, the expression of *IiDIR16* and *19* is induced by MeJA, this suggested that it is related to defense response (Li et al., 2014). Therefore, the function of PbDIRs in DIR-e remains to be further studied. The subfamily DIR-g is composed of PbDIRs and OsDIRs and the function of this subfamily members is not clear.

### Analysis of Protein Tertiary Structures and Key Amino Acid Residues

Phylogenetic tree analysis showed that only PbDIR4 and 5 were members of DIR-a subfamily. In this study, they were compared with 6 known functions of DIRs for protein tertiary structure analysis to understand its function.

**FIGURE 6:**
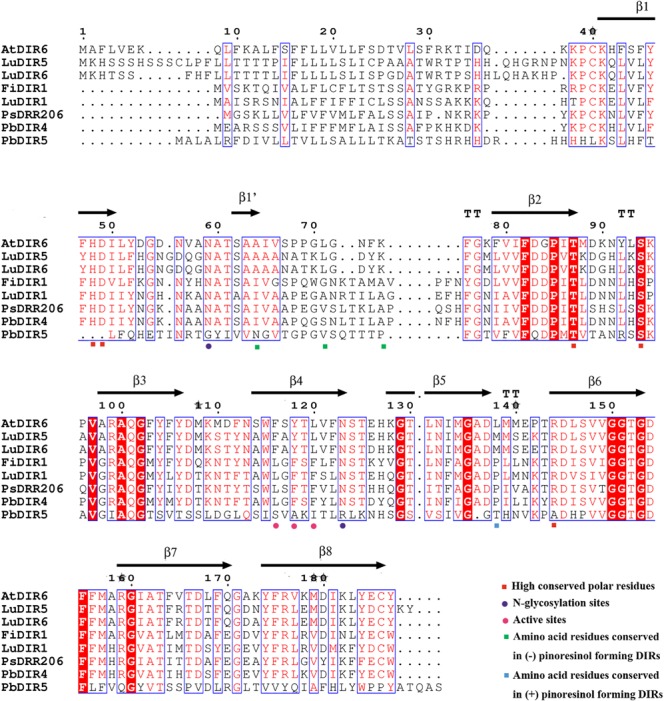
Amino acid sequence alignments of PbDIRs and other pinoresinol-forming DIRs.

At present, the crystal structure of AtDIR6 has been studied more clearly, so the name of each secondary structure, C terminus and N terminus of AtDIR6 are marked (Gasper et al., 2016). Prediction of protein tertiary structure found that the tertiary structure of each DIR-a subfamily [(+)-pinoresinol-forming DIR proteins: FiDIR1, LuDIR1, TpDIR8, TaDIR13, PsDRR206; (-)-pinoresinol-forming DIR protein: AtDIR6] have high similarity and are all β-barrel that composed of 8 β-sheets (β1, β1′, β2, β3, β4, β5, β6, β7, and β8), the direction and position of every β-sheet are very similar, there is also no significant difference in angles of C terminus and N terminus (Dalisay et al., 2015; Ma and Liu, 2015; Gasper et al., 2016). The tertiary structure of PbDIR4 is highly consistent with them and the key amino acid residue species is the same as (+)-pinoresinol-forming DIR proteins, suggesting that PbDIR4 can catalyze the generation of (+)-pinoresinol rather than (-)-pinoresinol and related to dimerization of monolignols. It is worth noting that the PbDIR5 of the same subfamily lacks β1′, and the direction and length of the C terminus domain are completely different from those of other DIR-a subfamilies (Supplementary Figure S5). Moreover, PbDIR5 lacks His^48^, Asp^49^, and Arg^144^ (**Figure 6**). These three residues are conserved in all characterized pinoresinol-forming DIRs and His^48^ is a key substrate binding site (Kim et al., 2012, 2015; Gasper et al., 2016; Paniagua et al., 2017). Two *N*-glycosylation sites also found that amino acid replacement (Gasper et al., 2016), indicating that deletion or alteration of the key amino acid residues of PbDIR5 resulted in a change in higher conformations that could eventually lead to a loss of catalytic function.

### Temporal Expression Analysis of *PbDIR*s

In order to clarify the expression patterns of *PbDIR* family members in ‘Dangshan Su’ pear fruits at different developmental stages, the qRT-PCR was used to analyze the expression level of these 35 *PbDIR*s at 8 different developmental stages of pear (15, 23, 39, 47, 63, 79, 102, and 145 DAF) and the results of qRT-PCR were presented in the form of heatmaps and the expression trends were clustered.

**FIGURE 7:**
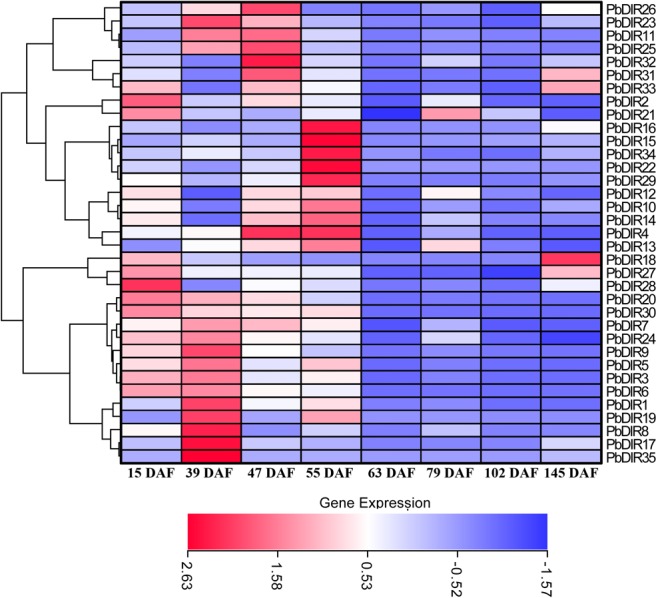
Expression profiles of *PbDIR* genes at different developmental stages of pear fruit.

It can be seen from **Figure 7** that the *PbDIR* family members are expressed in the fruit and reached the peak value at least for one period. Among them, *PbDIR15, 16, 22, 29*, and *34* had similar expression trends, and their expression reached the peak at 55 DAF, while at the other seven developmental stages, the expression levels were at a low level. *PbDIR1, 5, 8, 17, 19*, and *35* also had a similar expression pattern, which with a higher expression abundance at 39 DAF. In addition to *PbDIR5* and *19* with an expression peak at 55 DAF, the other four genes are expressed weakly after 39 DAF. The three genes (*PbDIR18, 27*, and *28*) were mainly expressed at the early stage of fruit development (15 DAF) or at maturity (145 DAF), and maintained at a low level during 39 to 102 DAF. *PbDIR11, 23, 25, 26, 31, 32*, and *33* were all highly expressed at 47 DAF. Among them, *PbDIR11, 23, 25*, and *26* also had high expression levels at 39 DAF, the expression of *PbDIR31*and *33* reached the second peak at 145 DAF. *PbDIR2* and *21* were mainly expressed at 15 DAF, and the expression of *PbDIR21* was up-regulated at 79 DAF. *PbDIR3, 6, 7, 9, 20, 24*, and *30* were mainly expressed at early stage of fruit development and decreased gradually after 39 DAF. *PbDIR4, 10, 12, 13*, and *14* were up-regulated at 47 and 55 DAF, but only *PbDIR4* was up-regulated at 47 and 55 DAF, and the expression of these five genes was low at 15, 39, 63, 79, 102, and 145 DAF. In addition, there are some genes that are up-regulated at maturity, such as the four genes *PbDIR18, 27, 31*, and *33*, and they may be involved in the synthesis of terpenoids of pear fruit to produce aroma (He et al., 2015; Effenberger et al., 2017; Paniagua et al., 2017).

### Hormone Response Pattern Analysis of *PbDIR*s

**FIGURE 8:**
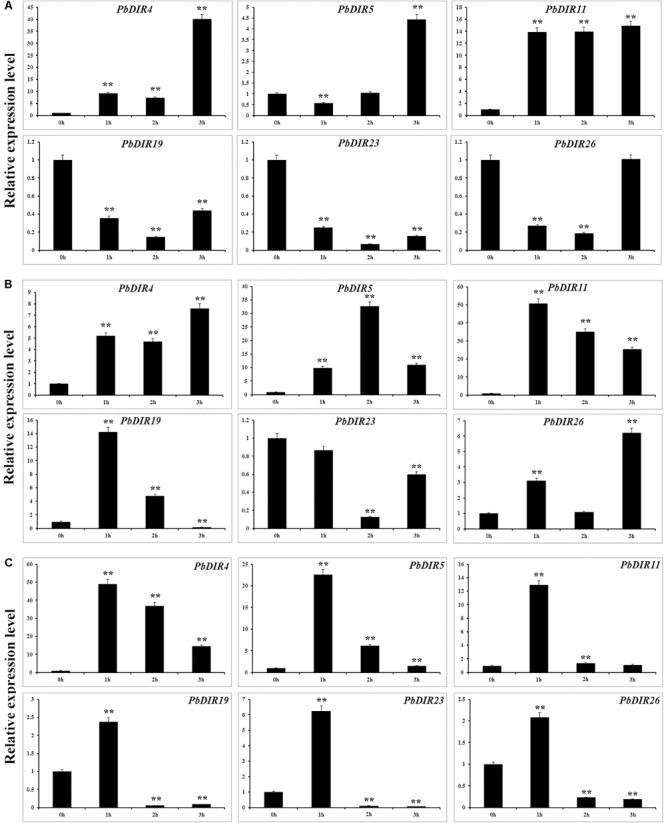
Hormone response pattern analysis of *PbDIR*s. *PbDIR*s expression in pear fruits in response to exogenous hormone (**A**: ABA; **B**: SA; **C**: MeJA) treatment for 0, 1, 2, and 3 h. Error bars indicate the standard deviation of three replications. Statistically analyzed using *t*-tests (^∗^*P* < 0.05, ^∗∗^*P* < 0.01).

In order to further analyze the response pattern of *PbDIR*s to hormones, we selected six genes from four subfamilies of *PbDIR* family and analyzed the response patterns with three hormones (ABA, MeJA, and SA) including *PbDIR4* and *5* of DIR-a, *PbDIR11* of DIR-b/d, *PbDIR19* of DIR-g, *PbDIR23* and *26* of DIR-e subfamily (**Figure 8**).

After the fruits treatment with ABA, these six *PbDIR*s showed two response patterns, one being induced by ABA, such as *PbDIR4, 5* and *11*; the other being inhibited by ABA, such as *PbDIR19, 23* and *26* (**Figure 8A**). Overall, except for *PbDIR5* showed a downward trend at 1 h post-treatment (HPT), the expression levels of these three genes (*PbDIR4, 5*, and *11*) were significantly increased at 1 to 3 HPT, and reached the peak at 3 HPT. However, it was completely different for *PbDIR19, 23*, and *26*. The expression levels of these three genes (*PbDIR19, 23*, and *26*) were significantly inhibited after ABA treatment, and the expression level was the lowest at 2 HPT. But the expression of *PbDIR26* almost recovered to its original level after treatment with ABA for 3 h.

*PbDIR4, 5, 11*, and *26* were up-regulated in SA-treated pear fruit. The expression of *PbDIR4* and *26* peaked at 3 HPT, *PbDIR5* peaked at 2 HPT, and the expression of *PbDIR11* reached the peak after treatment for 1 h. The expression pattern of *PbDIR19* was first induced and then inhibited after SA treatment, and which expression level reached a peak after treatment for 1 h, and significantly down regulated after treatment for 3 h. The expression of *PbDIR23* did not decrease significantly after treatment for 1 h, but decreased significantly at 2 and 3 HPT, and it was the lowest at 2 HPT (**Figure 8B**).

Methyl jasmonate treatment could significantly increase the expression of *PbDIR4, 5*, and *11* and reach the peak expression level when treated for 1 h. After treatment with MeJA for 1 h, *PbDIR19, 23*, and *26* were significantly induced, and then they also play a significant inhibitory effect on their expression for 2 and 3 h (**Figure 8C**).

## Discussion

The DIR and DIR-like play an important role in plant stress resistance and secondary metabolism. They can interact with laccase, mediate the polymerization and deposition of lignin in cell wall, and also be responsible for the dimerization of monolignols to form lignans resistant to pathogens (Lewis and Davin, 1998; Burlat et al., 2001; Kim et al., 2012; Shi et al., 2012; Barros et al., 2015; Paniagua et al., 2017). Although the *DIR* family has been screened and identified in some trees, medicinal plants and crops in recent years (Gasper et al., 2016), no studies have been reported on fruit trees, especially on pear. In this study, 35 *PbDIR*s were identified from the pear genome, and the bioinformatics analysis of this family was carried out. Meanwhile, *DIR* family members were also screened and identified from the peach, Mei, and grape genome. The evolutionary and collinearity relationship between *DIR*s of these four species was analyzed. Finally, through the temporal expression pattern analysis combined with phylogenetic tree, the tertiary structure of protein and the comparison of key amino acid residues to comparatively screen and identify the candidate members of lignin monomer polymerization.

It is found that the number of *PbDIR* family (35) is more than that of *DIR* family members were identified from the peach (29), Mei (26), and grape (14) genome. It may be that peach, Mei and grape only occurs once old whole-genome duplication (WGD) and pears not only occur once an old WGD but also a recent WGD (Jaillon et al., 2007; Zhang et al., 2012; Verde et al., 2013; Wu et al., 2013). We also identified five conserved characteristic motifs (Motif I-V) of the *PbDIR* family according to the method of Thamil Arasan et al. (2013) and Li et al. (2014) (Supplementary Figure S6). Multiple sequence alignment revealed that most of the PbDIRs contained these five motifs except for the deletion of individual members, which proved that the identification of *PbDIR* family members in this study was credible. However, Phe (F) in Motif-II mostly has been replaced by Ser (S) or Asn (N) in the PbDIRs of the DIR-e subfamily, but the Motif-II in *DIR* families of *I. indigotica* and Chinese cabbage is highly conserved. The Arg (R) and Glu (E) in Motif-V has been, respectively, replaced by Ser (S) and His (H) in the PbDIRs of the DIR-e subfamily, and a similar phenomenon in the *DIR* family of *I. indigotica*. The different kinds of amino acids in these sites may be related to the differentiation of different subfamily functions (Thamil Arasan et al., 2013; Li et al., 2014).

In order to better predict the function of PbDIRs, we constructed a phylogenetic tree between *PbDIR* family members and DIRs of other species. According to Ralph et al. (2007) classification method, members of *PbDIR* family is divided into four subfamilies (DIR-a, -b/d, -e, and -g) (**Figure 5**). Previous researches have suggested that DIR-a subfamily members are related to polymerization of lignin monomers (Kim et al., 2015; Ma and Liu, 2015; Gasper et al., 2016; Paniagua et al., 2017). However, in recent years it has also been found that some members of the DIR-b/d subfamily are involved in lignin or lignan metabolism, such as GmDIR22 and GhDIR1 (Shi et al., 2012; Li et al., 2017b). Shi et al. (2012) hold that GhDIR1 and 2 belong to DIR-e subfamily. But according to phylogenetic tree classification in this study, GhDIR1 and 2 should belong to DIR-b/d subfamily. The phylogenetic tree of Shi et al. (2012) with low bootstrap values, and AtDIR23 (DIR-b/d subfamily) are classified into DIR-e subfamily, so the classification result of this phylogenetic tree is inappropriate.

According to the results of phylogenetic tree clustering, PbDIR1, 2, 3, 4, and 5 may be involved in the formation of lignin or lignan (**Figure 5**). In which PbDIR4 and 5 belongs to the DIR-a subfamily and the expression level change trend is consistent with changes in lignin and stone cell content of fruits. However, due to the deletion and replacement of several key amino acid residues in PbDIR5, the catalytic activity has been lost or changed (**Figure 6**). Therefore, PbDIR4 is considered to be the bona fide DIR that catalyzes two coniferyl alcohol molecules to produce a pinoresinol and participates in the formation of lignin oligomers.

Cai et al. (2010) research has shown that the stone cells in ‘Dangshan Su’ pear were mainly formed from 23 to 67 DAF, and peaked at 55 DAF. Cheng et al. (2017) and Zhang et al. (2017) showed that the content of lignin in ‘Dangshan Su’ pear maintained a high level at 47 to 63 DAF, and the content of lignin and stone cells increased at first and then decreased at the whole developmental stage. The results of these 35 *PbDIR*s expression at eight developmental stages of fruit showed that the expression of *PbDIR4* was positively correlated with lignin and stone cells content peaks coincide, speculated that *PbDIR4* may be involved in stone cell development and lignin synthesis process. The expression of *PbDIR5* and *PbDIR19* peaked at 39 and 55 DAF, while lignin peaked at 47 and 63 DAF, respectively. These two genes peaked before lignin content peak, indicating that they may be involved in fruit lignin biosynthesis. The expression changes of *PbDIR1, 2*, and *3* were inconsistent with the expression changes of lignin and stone cells content in fruits. So although they were closely related to GhDIR1 and GmDIR22, they may not play a role in lignin synthesis and stone cell development, it is possible to participate in the synthesis of lignin or lignans in other tissues of pear (**Figure 7**).

In addition, the expression trend of five *DIR-like*s (DIR-b/d subfamily: *PbDIR11*; DIR-g subfamily: *PbDIR19*; DIR-e subfamily: *PbDIR23, 25*, and *26*) in fruits was also found to be related to the content changes of stone cells and lignin by qRT-PCR. Combined with the protein tertiary structure prediction, it was found that there was no β1′ in PbDIR11 and the C-terminal domain was significantly different from that of the pinoresinol-forming DIR proteins, which conformation is also different from GmDIR22 and GhDIR1. The tertiary structure of PbDIR19 is similar to that of GmDIR22, but it remains to be seen whether it has similar functions. The tertiary structures of PbDIR23, 25 and 26 are significantly different from those of pinoresinol-forming DIR proteins, suggesting that the functions of PbDIR23, 25, and 26 are likely to be different from them, but the structures of PbDIR23, 25, and 26 are similar to each other, and conforms to the classification of phylogenetic tree. At present, there are few studies on the function of the DIR-b/d subfamily and the function of the DIR-e and -g subfamilies is unknown (Supplementary Figure S7). Therefore, whether these five genes have the function of catalyzing lignin or lignan also have needs to be proved by enzymatic reactions *in vitro*, which will provide new insights into the function of DIR.

The analysis of the *cis*-elements in 5′ upstream region found that *PbDIR*s contain elements related to drought and light, indicating that drought and light may play a regulatory role in *PbDIR* (**Figure 4**). The development of stone cells in pear fruit is also regulated by water and light, speculated that *PbDIR* may play a role in it (Lee et al., 2006; Wang et al., 2017). In addition, a large number of elements responsive to ABA, SA and MeJA have been identified in 5′ upstream region of *PbDIR*s (Supplementary Table S5). We selected six *PbDIR*s from different subfamilies (DIR-b/d subfamily: *PbDIR11*; DIR-g subfamily: *PbDIR19*; DIR-e subfamily: *PbDIR23* and *26*) to study the hormone response patterns. For *PbDIR4*, which expression was higher at 1, 2, and 3 h after treatment with ABA, SA, and MeJA than that of untreated, indicating that its expression was induced by these three hormones, suggesting that it may play an important role in the biotic or abiotic stress of pear. The expression of *PbDIR5* and *PbDIR11* is very similar with *PbDIR4*, except that *PbDIR5* is inhibited after ABA treatment for 1 h. ABA inhibited the expression of *PbDIR23* and *PbDIR26*, whereas the expression of them was induced first and then inhibited with MeJA. SA inhibits the expression of *PbDIR23*, but induces the expression of *PbDIR26*. The *PbDIR19* of DIR-g subfamily was inhibited by ABA within 3 h treatment, while the expression patterns of MeJA and SA are first-induced and then inhibited (**Figure 8**). This indicates that the patterns of *PbDIR* response to hormones are quite complex. It is speculated that different *PbDIR*s play roles in different time periods in adversity environment.

## Conclusion

We identified 35 *PbDIR*s from the pear genome. Through systematic bioinformatics and qRT-PCR analysis, we concluded that PbDIR4 belongs to (+) pinoresinol-forming DIR protein and is also associated with the formation of stone cells and lignin in fruit. Which expression was significantly induced by SA, ABA, and MeJA, indicating that *PbDIR4* also plays a key role in biotic and abiotic stresses. In addition, we speculate that some *DIR-like*s (DIR-b/d subfamily: *PbDIR11*; DIR-g subfamily: *PbDIR19*; DIR-e subfamily: *PbDIR23, 25*, and *26*) are also likely involved in stone cell development and lignin metabolism of pear. This study not only clarified the molecular and evolutionary characteristics of the *PbDIR* family, but also lays the foundation for the further study on the functions of *PbDIR* family members, the regulation of the lignin polymerization and the development of stone cells in pear.

## Author Contributions

XC and XS performed the experiments and wrote the paper. XC and AM analyzed the data. AM and ML helped to polish the language. GL and YS helped to process the data. JZ and QJ contributed reagents and materials. XC and YC discussed and analyzed the results. YC and YL conceived and designed the experiments. All authors read and approved the final manuscript.

## Conflict of Interest Statement

The authors declare that the research was conducted in the absence of any commercial or financial relationships that could be construed as a potential conflict of interest.

## Footnotes

1 http://gigadb.org/dataset/100083

2 https://phytozome.jgi.doe.gov/pz/portal.html

3 http://pfam.xfam.org/

4 http://web.expasy.org/protparam/

5 http://www.genscript.com/wolf-psort.html

6 http://espript.ibcp.fr/ESPript/cgi-bin/ESPript.cgi

7 http://www.sbg.bio.ic.ac.uk/tildephyre2/html/page.cgi?id=index

8 http://gsds.cbi.pku.edu.cn/

9 http://meme-suite.org/

10 http://circos.ca/software/download/circos/

11 http://mapinspect.software.informer.com/

12 http://bioinformatics.psb.ugent.be/webtools/plantcare/html/

13 https://pypi.python.org/pypi/heatmapcluster
