# Supplementary material for: Molecular Characterization, Evolution, and Expression Profiling of the Dirigent (DIR) Family Genes in Chinese White Pear (Pyrus bretschneideri)
Source: Front Genet. 2018 Apr 16;9:136. doi: 10.3389/fgene.2018.00136 (PMC5911567; doi:10.3389/fgene.2018.00136)
Supplement: Supplementary file 1 [file Presentation_1.ZIP › Supplementary Figures.docx]

***Supplementary Material***

**Molecular characterization, evolution and expression profiling of the *Dirigent* (*DIR*) family genes in Chinese white pear (*Pyrus bretschneideri*)**

Xi Cheng^1^, Xueqiang Su^1^, Muhammad Abdullah^1^, Manli Li^1^, Jinyun Zhang^1,2^, Yanming Sun^1^, Guohui Li^1^, Qing Jin^1^, Yongping Cai^1*^, Yi Lin^1*^

^1^ School of Life Science, Anhui Agricultural University, No. 130, Changjiang West Road, Hefei 230036, China;

^2^ Horticultural Institute, Anhui Academy of Agricultural Sciences, Hefei, Anhui 230031, China;

^*^ Corresponding author:

Pro. Yongping Cai

ypcaiah@163.com (Y.C.)

Pro. Yi Lin

linyi1957@126.com (Y.L.)

**Supplementary Figures**


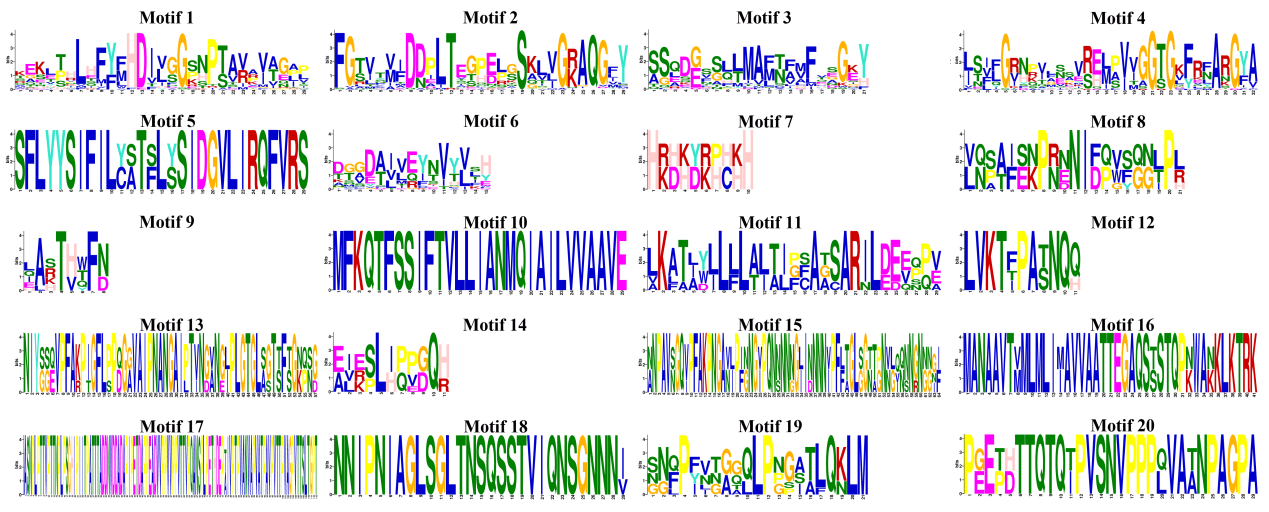


**Supplementary Figure 1 Motif composition of PbDIRs.**


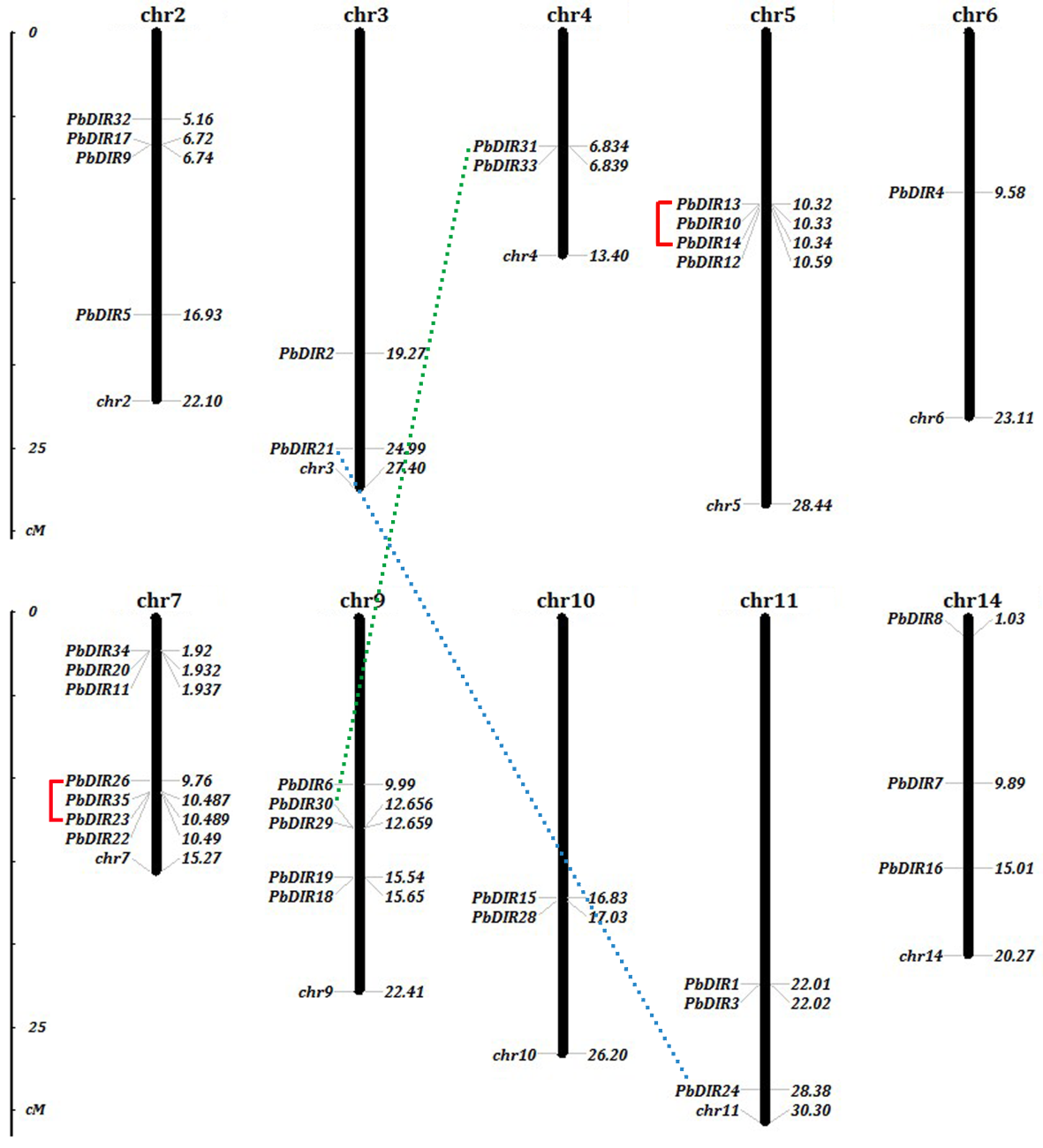


**Supplementary Figure 2 Chromosomal locations and gene duplications of *PbDIR*s on ten chromosomes.** Genes involved in tandem duplication are joined by red lines. Genes involved in segmental duplication are joined by dotted lines.

**
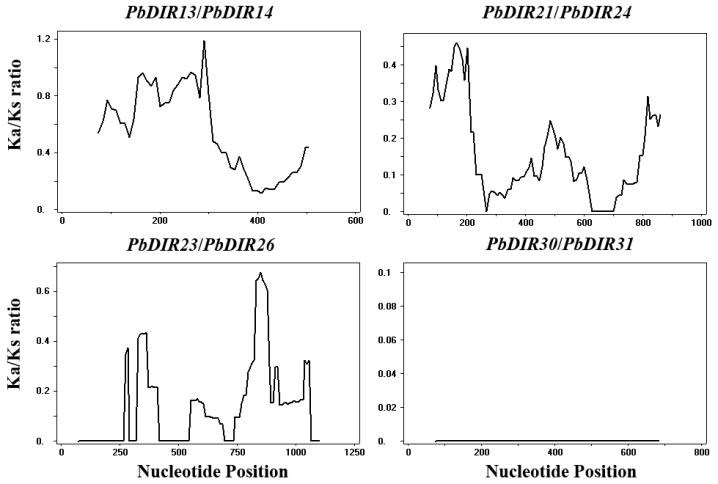
**

**Supplementary Figure 3 Sliding window plots of duplicated *PbDIR*s.** The window size was 150 bp, and the step size was 9 bp. The X-axis represents the nucleotide position of the gene pair,  and the Y-axis represents the ratio of Ka/Ks.

**Supplementary Figure
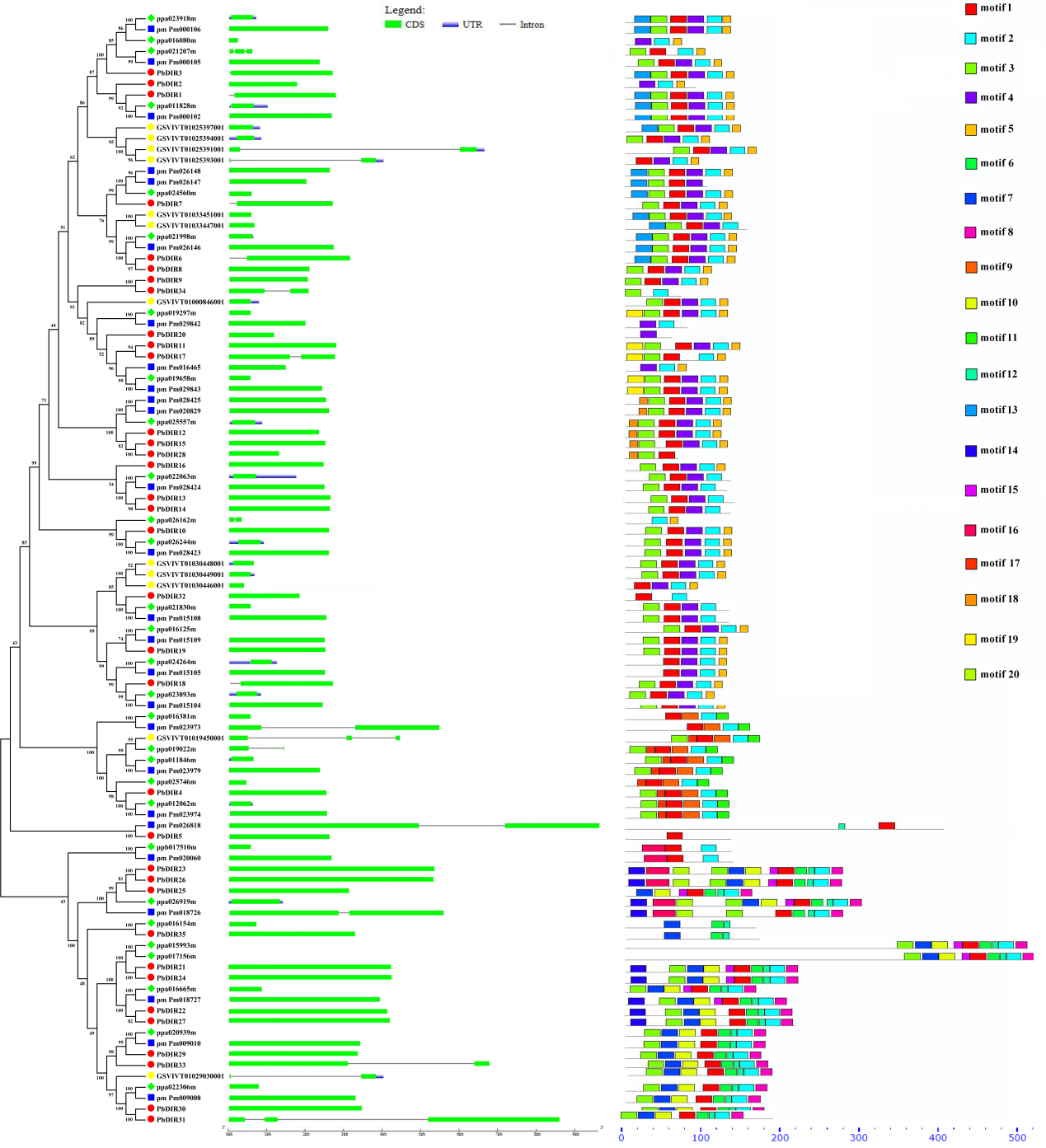
4 Neighbor-Joining tree, gene structures and conserved motifs of *DIR* family members in four species.** *V*. *vinifera* (yellow), *P*. *mume* (blue), *P*. *persica* (green), and *P*. *bretschneideri* (red).

**
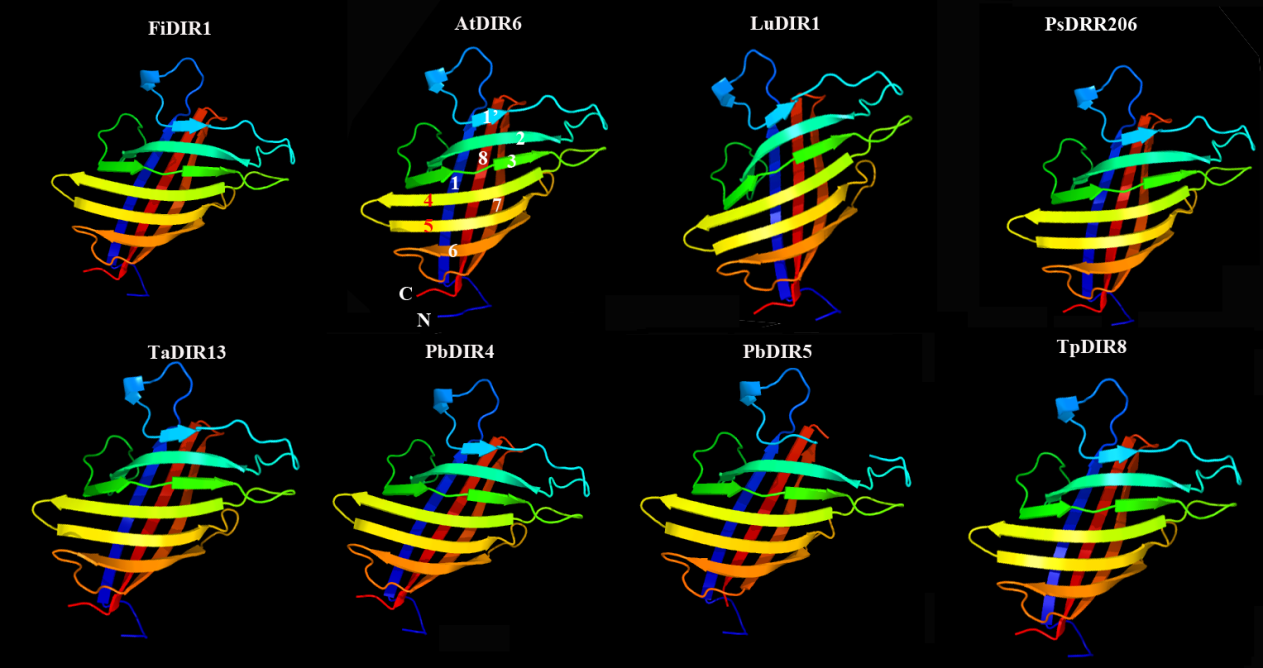
**

**Supplementary Figure 5 Protein tertiary structures of PbDIRs from DIR-a subfamily and other pinoresinol-forming DIRs.** 1~8: eight β-sheets; N terminus and C terminus are colored blue and red.


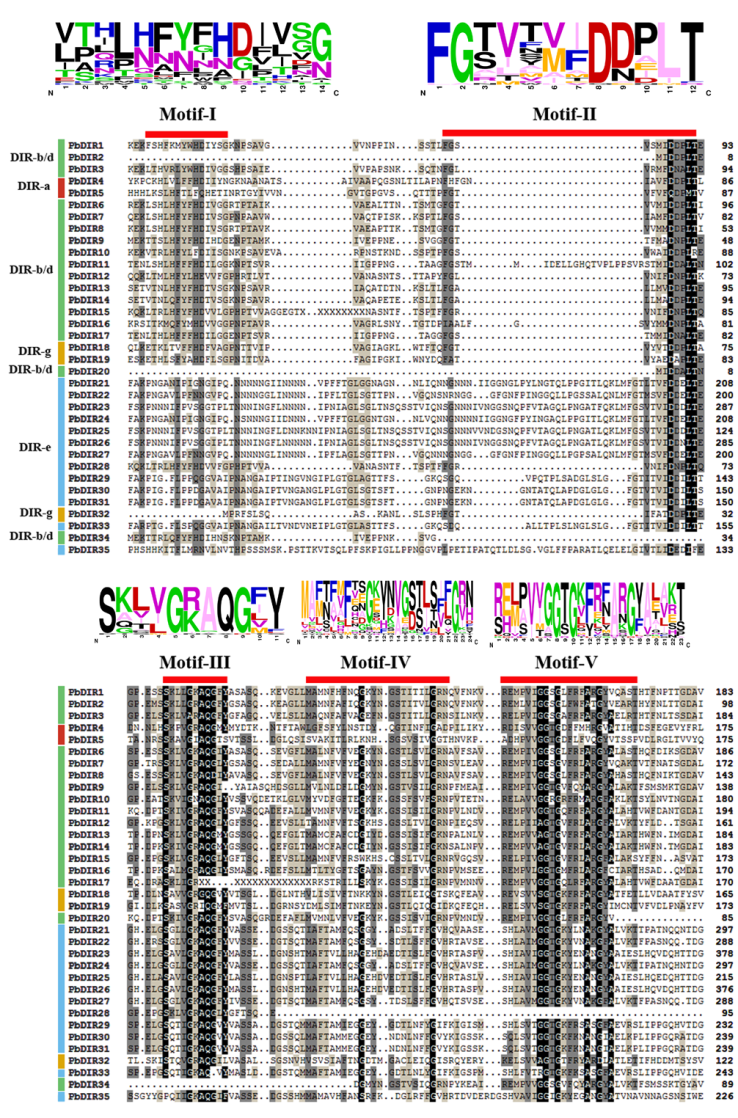


**Supplementary Figure 6 Conserved five characteristic motifs of PbDIRs.**

**
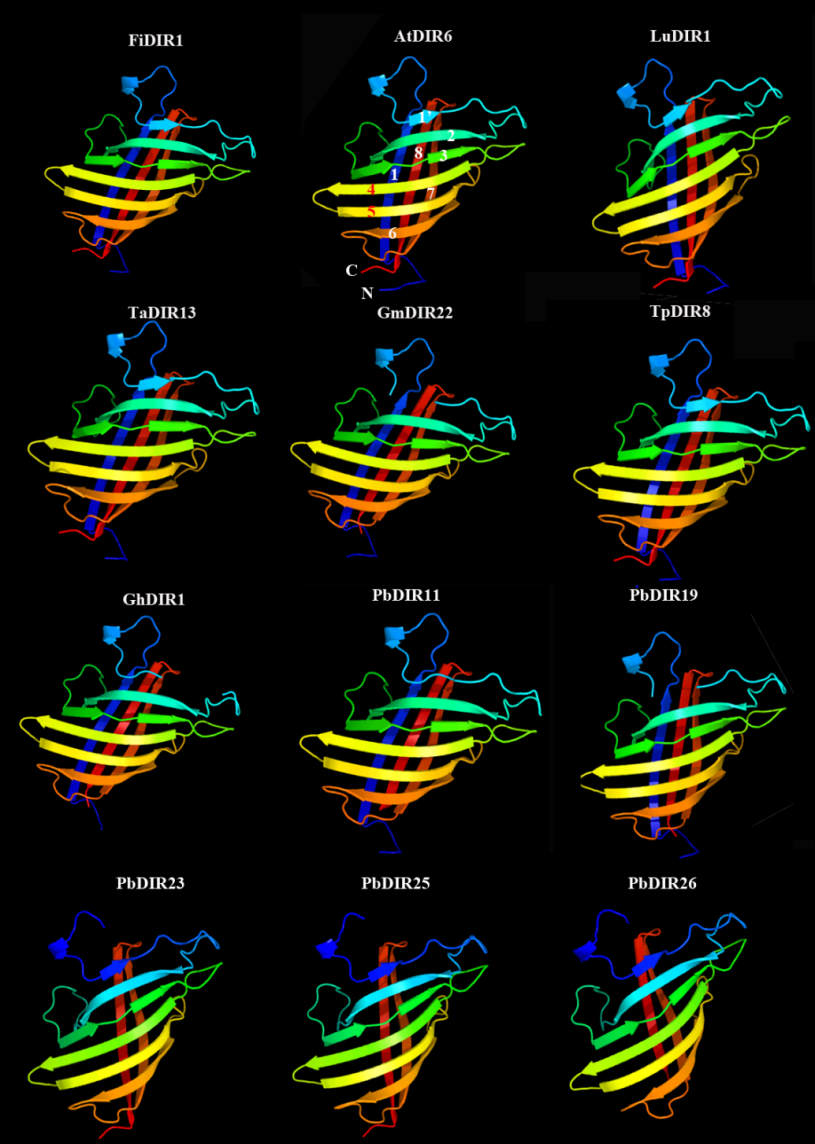
**

**Supplementary Figure 7 Protein tertiary structures of PbDIRs from DIR-b/d, -g, -e subfamily and other pinoresinol-forming DIRs.** 1~8: eight β-sheets; N terminus and C terminus are colored blue and red.
