# Supplementary material for: Molecular Characterization, Evolution, and Expression Profiling of the Dirigent (DIR) Family Genes in Chinese White Pear (Pyrus bretschneideri)
Source: Front Genet. 2018 Apr 16;9:136. doi: 10.3389/fgene.2018.00136 (PMC5911567; doi:10.3389/fgene.2018.00136)
Supplement: Supplementary file 1 [file Presentation_1.ZIP › Supplementary Tables.doc]

***Supplementary Material***

**Molecular characterization, evolution and expression profiling of the *Dirigent* (*DIR*) family genes in Chinese white pear (*Pyrus bretschneideri*)**

Xi Cheng^1^, Xueqiang Su^1^, Muhammad Abdullah^1^, Manli Li^1^, Jinyun Zhang^1,2^, Yanming Sun^1^, Guohui Li^1^, Qing Jin^1^, Yongping Cai^1*^, Yi Lin^1*^

^1^	School of Life Science, Anhui Agricultural University, No. 130, Changjiang West Road, Hefei 230036, China;

^2^	Horticultural Institute, Anhui Academy of Agricultural Sciences, Hefei, Anhui 230031, China;

^*^ Corresponding author:

Pro. Yongping Cai

ypcaiah@163.com (Y.C.)

Pro. Yi Lin

linyi1957@126.com (Y.L.)

**Supplementary Tables**

**Supplementary Table 1 Gene accession codes used for constructing phylogenetic trees.**

| **Gene Name** | **Gene No.** | **Species** | **Gene Name** | **Gene No.** | **Species** | |
| --- | --- | --- | --- | --- | --- | --- |
| ***AtDIR1*** | At5g42510 | *Arabidopis thaliana* | ***GhDIR1*** | ACU55135 | *Gossypiumhirsutum* |  |
| ***AtDIR2*** | At5g42500 | *Arabidopis thaliana* | ***GhDIR2*** | ACU55136 | *Gossypiumhirsutum* |  |
| ***AtDIR3*** | At5g49040 | *Arabidopis thaliana* | ***TaDIR13*** | TC299725 | *Triticumaestivum* |  |
| ***AtDIR4*** | At2g21110 | *Arabidopis thaliana* | ***IiDIR1*** | Ii13356c0 | *Isatisindigotica* |  |
| ***AtDIR5*** | At1g64160 | *Arabidopis thaliana* | ***IiDIR2*** | Ii33656c0 | *Isatisindigotica* |  |
| ***AtDIR6*** | At4g23690 | *Arabidopis thaliana* | ***IiDIR3*** | Ii22738c0 | *Isatisindigotica* |  |
| ***AtDIR7*** | At3g13650 | *Arabidopis thaliana* | ***IiDIR4*** | Ii22738c0 | *Isatisindigotica* |  |
| ***AtDIR8*** | At3g13662 | *Arabidopis thaliana* | ***IiDIR5*** | Ii20562c1 | *Isatisindigotica* |  |
| ***AtDIR9*** | At2g39430 | *Arabidopis thaliana* | ***IiDIR6*** | Ii20562c1 | *Isatisindigotica* |  |
| ***AtDIR10*** | At2g28670 | *Arabidopis thaliana* | ***IiDIR7*** | Ii28741c0 | *Isatisindigotica* |  |
| ***AtDIR11*** | At1g22900 | *Arabidopis thaliana* | ***IiDIR8*** | Ii28741c0 | *Isatisindigotica* |  |
| ***AtDIR12*** | At4g11180 | *Arabidopis thaliana* | ***IiDIR9*** | Ii29140c0 | *Isatisindigotica* |  |
| ***AtDIR13*** | At4g11190 | *Arabidopis thaliana* | ***IiDIR10*** | Ii33689c0 | *Isatisindigotica* |  |
| ***AtDIR14*** | At4g11210 | *Arabidopis thaliana* | ***IiDIR11*** | Ii29669c0 | *Isatisindigotica* |  |
| ***AtDIR15*** | At4g38700 | *Arabidopis thaliana* | ***IiDIR12*** | Ii32977c0 | *Isatisindigotica* |  |
| ***AtDIR16*** | At3g24020 | *Arabidopis thaliana* | ***IiDIR13*** | Ii26249c0 | *Isatisindigotica* |  |
| ***AtDIR17*** | AT5G49030 | *Arabidopis thaliana* | ***IiDIR14*** | Ii26249c0 | *Isatisindigotica* |  |
| ***AtDIR18*** | At4g13580 | *Arabidopis thaliana* | ***IiDIR15*** | Ii26249c0 | *Isatisindigotica* |  |
| ***AtDIR19*** | At1g58170 | *Arabidopis thaliana* | ***IiDIR16*** | Ii14231c0 | *Isatisindigotica* |  |
| ***AtDIR20*** | At1g55210 | *Arabidopis thaliana* | ***IiDIR17*** | Ii32662c0 | *Isatisindigotica* |  |
| ***AtDIR21*** | At1g65870 | *Arabidopis thaliana* | ***IiDIR18*** | Ii18321c3 | *Isatisindigotica* |  |
| ***AtDIR22*** | At3g13660 | *Arabidopis thaliana* | ***IiDIR19*** | Ii30687c1 | *Isatisindigotica* |  |
| ***AtDIR23*** | At2g21100 | *Arabidopis thaliana* | ***FiDIR1*** | AAF25357 | *Forsythia intermedia* |  |
| ***AtDIR24*** | At3g55230 | *Arabidopis thaliana* | ***FiDIR2*** | AAF25358 | *Forsythia intermedia* |  |
| ***AtDIR25*** | AT1G07730 | *Arabidopis thaliana* | ***TpDIR5*** | AF210067 | *Thujaplicata* |  |
| ***GmDIR22*** | HQ993047 | *Glycine max* | ***TpDIR8*** | AF210070 | *Thujaplicata* |  |

**Supplementary Table 2 Primers for qRT-PCR.**

| **Name** | **Sense primer sequence (5’-3’)** | **Anti-sense primer sequence (5’-3’)** |
| --- | --- | --- |
| **PbDIR1** | ACTCATACATTCAATCCAACCACAG | AACAAAGCAAAAGCCATCAAGAAAA |
| **PbDIR2** | ATGTTGAAGCAAGAACTCACTATTT | AGACCTTAAAACAAAAGCAAGCTAG |
| **PbDIR3** | TTGGTGGAAGTGGGGCTTTCAGGTT | GGCCAAATCAAATTCCAAAACCCAT |
| **PbDIR4** | TCAATATTAAACAAGGTGAGGGAGC | TACAACCAAGCAATGGCCAAATCAA |
| **PbDIR5** | CCTAACTGTTGTCTACCAGATTGCA | TGGAGATACAAAATCACACTTAAGA |
| **PbDIR6** | AAAGTGGAGATGCTGTTG | AAAGCTAGGAAAACAAACAAC |
| **PbDIR7** | GATGCTCTTGTTGAATATGATGT | CTACACTTTGACCGAAGACA |
| **PbDIR8** | ACTGGAGATGCTGTTGTGGAAT | TAGGAAAGCAAATAACACGCACAC |
| **PbDIR9** | CTGGAGATGCTGTTGTTG | CTCTGATTGAGATGATATGATACA |
| **PbDIR10** | GGTGATGCCATTATTGAGTAT | CTTCATTACAGTTGCAGGAT |
| **PbDIR11** | GATGCTAATACGGGAGAT | TTTCTCAACAAATGGCTAG |
| **PbDIR12** | CTAGTGGTGCCATTGTTGAAT | GGAACCCACACGATTTACAC |
| **PbDIR13** | ATTATGGGTGATGCAATT | AATCACAAGTGTACTAGC |
| **PbDIR14** | GGTTTAATACTATGGGTGAT | GATCTCATTAGTGTACTAGC |
| **PbDIR15** | GTTGCCACTGTTGAATATAATG | GGATACACCAGAAACATATCTTAA |
| **PbDIR16** | ATTCTGCGGACCAAATGG | ACGGTATGATCTAATTAATCTGAG |
| **PbDIR17** | GATGCTGCTACAGGAGAT | TTTTCTCAACAATGGCTAG |
| **PbDIR18** | TGCTGCAACTTTCTACTC | ATGGCATCAACACAGAGA |
| **PbDIR19** | GACCTTCCAAATGCATACTTT | ACCAGTAGTGTTTCATCATAGA |
| **PbDIR20** | TTAGGTTTGCTCGTGGCTAT | TACTCAACAATGGCATCTCCT |
| **PbDIR21** | CAGCAAAACACTGATGGA | AAAACCTTAATTAGCACAGTAAG |
| **PbDIR22** | AGACGCTGTTGGAGTTCA | AAACTTGAAACACAAATGCTCTC |
| **PbDIR23** | GGATACCATTATGCAGATCAG | TCTCACAACCCTAACTGG |
| **PbDIR24** | CAGCACAACACTGATGGA | AAAACCTTAATTAGCACAGTAAG |
| **PbDIR25** | GGGTGGATACTATTATGC | GTACATCAAATGCACATT |
| **PbDIR26** | GACCAGCACACGACTGAT | AAACATACCACCACTTTCTATTCA |
| **PbDIR27** | AGACGCTGTTGCAGTTCA | GCAGACCTTACAAACAAACTTGA |
| **PbDIR28** | CCTGGTTCCAAGCTCGTG | AAACAAAGTTCATAGCCATAAGCA |
| **PbDIR29** | CTGATGGGGCTGAGACTTT | AGTAAATCTCCACAACACTGAATT |
| **PbDIR30** | TCAACGAGCAACAGATGG | GCACAACAGACCAGACAT |
| **PbDIR31** | GTGATGGATCGTGAAGAG | GGAGATTACTTAATCAATCAATC |
| **PbDIR32** | ATATGACCTCCTACTCAGT | GATGTTGAAGCGACTACT |
| **PbDIR33** | TGTCATTGATGAGGCTGAG | AAATCTCCACACCATCAAATTC |
| **PbDIR34** | GGTGAAGACATTCTCGAT | CCATCAAATTCTCAGTAGTG |
| **PbDIR35** | CTATCTGGGAAGAAGAAAACA | GTTTCGACGTAACAAGGT |

**Supplementary Table 3 Ka/Ks analysis for *PbDIR* duplicated genes of pear.**

| **Duplicated gene pairs** | | **Ka** | **Ks** | **Ka/Ks** | **Purifying selection** | **Duplicated type** |
| --- | --- | --- | --- | --- | --- | --- |
| ***PbDIR30*** | ***PbDIR31*** | **0.0089** | **0.0105** | **0.8476** | **Yes** | **Segmental** |
| ***PbDIR23*** | ***PbDIR26*** | **0.0082** | **0.0376** | **0.2181** | **Yes** | **Tandem** |
| ***PbDIR21*** | ***PbDIR24*** | **0.0410** | **0.2351** | **0.1744** | **Yes** | **Segmental** |
| ***PbDIR13*** | ***PbDIR14*** | **0.0656** | **0.1184** | **0.5541** | **Yes** | **Tandem** |

**Supplementary Table 4** Sequence information of PmDIRs, VvDIRs and PpDIRs.

| **Sequence ID** | **Chr** | **Protein** | | |
| --- | --- | --- | --- | --- |
|  |  | **Length (aa)** | **MW**  **(kDa)** | **pI** |
| Pm026146 | 8 | 199 | 21.9 | 9.13 |
| Pm000102 | 1 | 194 | 21.4 | 9.15 |
| Pm026148 | 8 | 191 | 20.8 | 9.77 |
| Pm000105 | 1 | 172 | 19.0 | 7.07 |
| Pm029843 | scaffold364 | 182 | 20.0 | 6.18 |
| Pm028425 | scaffold126 | 189 | 20.7 | 8.98 |
| Pm020829 | 6 | 189 | 20.7 | 8.98 |
| Pm028424 | scaffold126 | 181 | 19.4 | 8.67 |
| Pm000106 | 1 | 188 | 20.5 | 8.96 |
| Pm028423 | scaffold126 | 189 | 20.7 | 9.30 |
| Pm026147 | 8 | 146 | 15.7 | 9.75 |
| Pm016465 | 5 | 108 | 11.9 | 4.93 |
| Pm015105 | 4 | 181 | 19.5 | 4.88 |
| Pm015104 | 4 | 177 | 19.6 | 6.91 |
| Pm015109 | 4 | 181 | 19.7 | 6.40 |
| Pm015108 | 4 | 184 | 20.0 | 8.73 |
| Pm029842 | scaffold364 | 111 | 12.2 | 6.72 |
| Pm023979 | 7 | 173 | 19.4 | 8.61 |
| Pm023974 | 7 | 185 | 20.7 | 8.83 |
| Pm023973 | 7 | 221 | 24.8 | 8.74 |
| Pm018727 | 5 | 286 | 30.2 | 4.65 |
| Pm009010 | 2 | 249 | 25.5 | 5.60 |
| Pm018726 | 5 | 387 | 41.3 | 5.20 |
| Pm009008 | 2 | 240 | 24.5 | 5.41 |
| Pm020060 | 6 | 194 | 21.0 | 7.95 |
| Pm026818 | 8 | 567 | 62.9 | 8.75 |
| GSVIVT01033451001 | 8 | 189 | 20.7 | 9.19 |
| GSVIVT01033447001 | 8 | 216 | 23.8 | 8.96 |
| GSVIVT01000846001 | 13 | 182 | 19.9 | 6.65 |
| GSVIVT01025397001 | 6 | 205 | 23.1 | 9.47 |
| GSVIVT01025391001 | 6 | 233 | 26.0 | 8.93 |
| GSVIVT01025394001 | 6 | 149 | 16.1 | 5.59 |
| GSVIVT01025393001 | 6 | 131 | 14.3 | 8.73 |
| GSVIVT01030448001 | 12 | 177 | 19.5 | 8.69 |
| GSVIVT01030449001 | 12 | 179 | 19.7 | 7.85 |
| GSVIVT01030446001 | 12 | 127 | 14.2 | 9.00 |
| GSVIVT01019450001 | 2 | 239 | 27.2 | 9.69 |
| GSVIVT01005968001 | / | 323 | 33.2 | 4.97 |
| GSVIVT01029030001 | 5 | 255 | 26.1 | 6.19 |
| GSVIVT01033453001 | 8 | 132 | 15.1 | 10.88 |
| ppa021998m | 7 | 198 | 21.8 | 7.11 |
| ppa011828m | 6 | 194 | 21.4 | 9.33 |
| ppa024560m | 7 | 191 | 20.7 | 9.40 |
| ppa019658m | 2 | 182 | 20.0 | 6.18 |
| ppa019297m | 2 | 183 | 20.5 | 9.15 |
| ppa025557m | 8 | 171 | 18.7 | 8.09 |
| ppa023918m | 6 | 188 | 20.4 | 9.22 |
| ppa022063m | 8 | 189 | 20.2 | 8.66 |
| ppa026244m | 8 | 189 | 20.8 | 9.48 |
| ppa021207m | 6 | 142 | 15.8 | 6.56 |
| ppa023893m | 3 | 157 | 17.4 | 6.47 |
| ppa024264m | 3 | 181 | 19.4 | 4.89 |
| ppa016080m | 6 | 101 | 11.0 | 7.31 |
| ppa016125m | / | 219 | 24.1 | 6.65 |
| ppa021830m | 3 | 184 | 19.9 | 8.73 |
| ppa011846m | 5 | 193 | 21.5 | 8.59 |
| ppa015993m | / | 714 | 78.9 | 7.23 |
| ppa017156m | / | 727 | 80.5 | 7.00 |
| ppa026919m | 2 | 420 | 42.9 | 4.61 |
| ppa016665m | 2 | 232 | 24.3 | 4.84 |
| ppa012062m | 5 | 185 | 20.7 | 8.73 |
| ppa019022m | 5 | 165 | 18.4 | 9.56 |
| ppa020939m | 1 | 249 | 25.5 | 6.03 |
| ppa016381m | 5 | 184 | 20.7 | 8.43 |
| ppa022306m | 1 | 252 | 25.9 | 5.64 |
| ppa025746m | 5 | 149 | 16.6 | 5.44 |
| ppa016154m | 2 | 232 | 25.0 | 6.29 |
| ppa017510m | 8 | 190 | 20.5 | 7.93 |
| ppa026162m | 8 | 94 | 10.5 | 9.37 |

Note: “/” indicates that the specific chromosome is not known.

**Supplementary Table 5 Numbers of cis-elements in the 5' upstream region of *PbDIR*s.**

| Elements | MBS | MRE | LTR | HSE | TC-rich repeat | ABRE | ERE | CGTCA motif | TCA-element | Total |
| --- | --- | --- | --- | --- | --- | --- | --- | --- | --- | --- |
| Function | Drought stress | Light response | Low-temperature stress | Heat stress | Defense and stress | ABA response | Ethylene response | MeJA response | SA response |  |
| *PbDIR1* | 2 |  | 1 | 1 |  | 1 | 1 | 1 | 2 | 12 |
| *PbDIR2* | 2 | 2 | 1 |  |  |  | 1 |  | 2 | 9 |
| *PbDIR3* | 2 | 1 | 2 |  | 2 | 1 |  | 1 | 1 | 13 |
| *PbDIR4* |  | 1 |  | 2 | 1 | 1 | 1 |  | 1 | 7 |
| *PbDIR5* | 2 |  |  |  |  | 1 |  |  | 3 | 6 |
| *PbDIR6* | 1 |  | 1 | 1 | 2 | 2 |  | 3 |  | 10 |
| *PbDIR7* | 3 |  |  |  |  |  | 1 | 3 |  | 7 |
| *PbDIR8* |  |  | 1 | 1 | 4 |  |  |  | 1 | 8 |
| *PbDIR9* | 2 |  |  | 3 | 1 | 2 |  |  |  | 8 |
| *PbDIR10* |  |  |  |  |  | 1 |  | 1 | 1 | 5 |
| *PbDIR11* |  |  | 3 | 3 | 1 | 2 | 2 |  |  | 11 |
| *PbDIR12* | 1 |  | 2 | 3 | 1 | 2 |  | 2 | 1 | 12 |
| *PbDIR13* | 4 | 2 | 2 |  | 1 | 1 |  |  | 2 | 12 |
| *PbDIR14* | 1 | 1 | 1 |  | 2 |  | 2 | 2 | 1 | 10 |
| *PbDIR15* | 1 |  |  |  |  |  |  |  | 4 | 5 |
| *PbDIR16* | 2 | 1 |  |  | 2 | 1 | 1 | 1 | 2 | 10 |
| *PbDIR17* | 3 |  | 2 | 4 | 1 |  |  |  | 2 | 12 |
| *PbDIR18* | 3 | 1 |  | 1 |  |  |  | 1 |  | 6 |
| *PbDIR19* | 1 |  |  |  | 1 |  |  | 3 | 2 | 7 |
| *PbDIR20* | 1 | 1 | 1 |  |  | 1 |  | 2 | 1 | 7 |
| *PbDIR21* | 1 | 1 |  |  | 1 |  |  | 1 |  | 4 |
| *PbDIR22* | 2 |  | 2 | 1 |  | 1 | 1 | 5 |  | 12 |
| *PbDIR23* | 2 |  |  |  | 1 |  |  | 2 | 1 | 6 |
| *PbDIR24* | 1 |  |  | 1 | 3 |  |  | 1 | 1 | 7 |
| *PbDIR25* | 4 | 1 |  |  | 1 |  |  | 2 |  | 10 |
| *PbDIR26* | 2 |  |  |  | 1 |  |  | 2 | 1 | 6 |
| *PbDIR27* | 2 |  | 1 | 2 | 1 | 2 |  | 2 |  | 10 |
| *PbDIR28* | 1 |  |  | 1 |  |  |  | 2 | 4 | 8 |
| *PbDIR29* | 2 |  | 1 | 2 |  | 2 |  | 1 | 1 | 9 |
| *PbDIR30* | 2 |  | 1 | 2 |  | 1 |  | 3 | 1 | 10 |
| *PbDIR31* | 2 |  | 1 | 2 |  | 1 |  | 3 | 1 | 10 |
| *PbDIR32* |  |  | 2 | 1 |  | 6 |  | 1 |  | 10 |
| *PbDIR33* | 2 |  | 2 | 4 | 2 | 2 | 1 | 3 |  | 16 |
| *PbDIR34* | 1 |  |  | 1 | 1 | 3 |  | 1 | 2 | 9 |
| *PbDIR35* | 2 |  | 1 | 1 |  |  |  | 2 | 2 | 8 |
| Total | 57 | 12 | 28 | 37 | 30 | 34 | 11 | 51 | 40 | 312 |
